# Supplementary material for: Integrated Proteomics and Metabolomics Link Acne to the Action Mechanisms of Cryptotanshinone Intervention
Source: Front Pharmacol. 2021 Sep 1;12:700696. doi: 10.3389/fphar.2021.700696 (PMC8440807; doi:10.3389/fphar.2021.700696)
Supplement: Supplementary file 1 [file DataSheet1.ZIP › Supplementary table 1-10 and material 1-3/Material 3-Metlib-MSMS/POS-Metlib-MSMS/Metlib-MSMS/M309T36_forward/0.684,Retinol (Vitamin A),(M+Na)+.pdf]

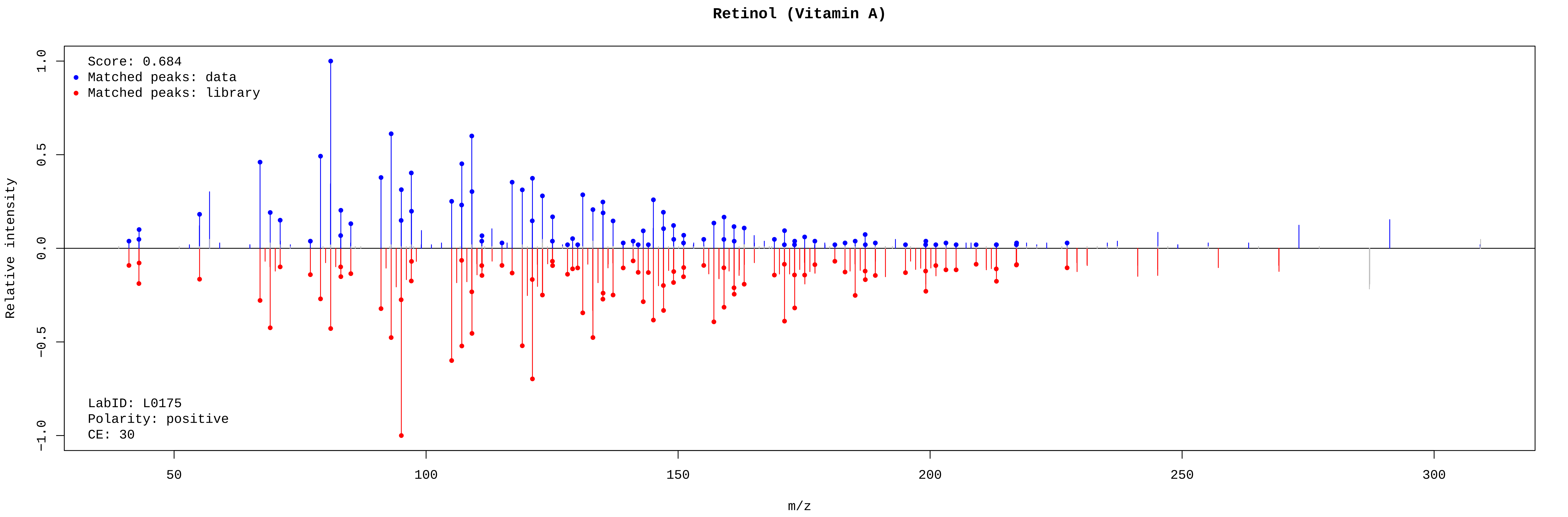
Retinol (Vitamin A)
Score: 0.684
Matched peaks: data
Matched peaks: library
LabID: L0175
Polarity: positive
CE: 30
Relative intensity
m/z
1.0
0.5
0.0
-0.5
-1.0
50
100
150
200
250
300
